# What’s in a name? Refining the nomenclature of liver metastases growth patterns by changing “desmoplastic” to “encapsulated”

**DOI:** 10.1038/s44276-023-00018-6

**Published:** 2023-12-12

**Authors:** Carlos Fernández Moro, Béla Bozóky, Natalie Geyer, Jennie Engstrand, Luc Dirix, Peter Vermeulen, Marco Gerling

**Affiliations:** 1 Department of Biosciences and Nutrition, Karolinska Institutet, 14183 Huddinge, Sweden; 2 Department of Clinical Pathology and Cancer Diagnostics, Karolinska University Hospital, Stockholm, 14186 Sweden; 3 Department of Laboratory Medicine, Division of Pathology, Karolinska Institutet, 14186 Stockholm, Sweden; 4 Department of Clinical Science, Intervention and Technology, Division of Surgery, Karolinska Institutet, Karolinska University Hospital, 14152 Stockholm, Sweden; 5 Translational Cancer Research Unit (GZA Hospitals and University of Antwerp), Antwerp, Belgium; 6 Theme Cancer, Karolinska University Hospital, 17 176 Solna, Sweden

**arising from** E. Latacz et al. *. Br J Cancer* 10.1038/s41416-022-01859-7 (2022)

Liver metastases growth patterns are conventionally classified as either “replacement” or “desmoplastic”. We and others have suggested this classification in the latest international consensus guidelines recently published in the British Journal of Cancer [1]. While other rare patterns exist, “replacement” and “desmoplastic” are the most common patterns. They are associated with clear differences in survival, such that predominantly desmoplastic colorectal cancer metastases have a more favorable liver relapse-free and overall survival, as demonstrated in large international, retrospective studies [1, 2].

The growth patterns are currently not a part of the routine assessment of liver metastases in most pathology departments. Because they outperform other known prognostic markers [1], we hope and actively work for them to be implemented in routine diagnostics. Given their potential clinical implementation, it will be important to carefully reassess the growth patterns’ nomenclature to avoid misunderstandings. Against this background, we appreciate the suggestion by Kong and colleagues to change the term “replacement” to “infiltrative” [3]. Nevertheless, we argue for keeping the current term, “replacement” because during infiltration into the liver, cancer cells morphologically indeed co-opt the sinusoidal blood vessels by taking the place of the hepatocytes as outlined previously [4].

However, we here suggest changing the term for “desmoplastic” to “encapsulated”, based on the careful reassessment of the connotations of both terms and in light of new experimental data. The name change serves two purposes: 1. It avoids confusion with a common connotation of “desmoplasia,” which refers to stromal activation, associated with aggressive tumor invasion; 2. It takes into account new experimental evidence that links the formation of the perimetastatic capsule to a benign-like fibrotic reaction as part of a reparative hepatic process.

Firstly, “encapsulated” avoids conflation with the so-called “desmoplastic” stroma, which has more commonly been associated with aggressive tumor growth, most prominently in pancreatic ductal adenocarcinoma [5], but also in other malignancies, such as primary colorectal cancer [6]. Although the term “desmoplasia” is loosely defined in the literature and may refer to any pathological stroma regardless of the mechanism, many physicians and researchers may perceive and interpret ‘desmoplasia’ as the result of active, aggressive tumor growth; this prevailing view is supported by studies that have shown negative correlations between the amount of desmoplasia and tumor-related outcomes [5, 6]. The connection of negative outcomes with “desmoplasia” is at odds with the fibrotic capsule seen in patients with liver metastases, because the stromal rim in liver metastases is associated with a significant survival advantage [1]. We acknowledge that the protective effects of the desmoplastic intratumoral stroma are being increasingly recognized. Nevertheless, because we and others in the field aim at the clinical implementation of the growth patterns, we argue for a terminology that avoids the possibilty of confounding the perimetastatic rim with intratumoral desmoplasia, which would be achieved by using “encapsulated” instead.

Recent functional evidence from our group has shed light on the processes underlying capsule formation [7]. We have analyzed human colorectal cancer liver metastases and found remnants of liver parenchyma predominantly in the outer zone of the rim. Protein profiling revealed a zonation of the rim, such that benign-like stroma markers were seen in the outer part of the rim. In contrast, the tumor-adjacent inner rim showed similarities to the stroma of the metastasis centrum. We also observed a strong positive correlation between neoadjuvant chemotherapy and the presence of the capsule, which had been described before in prospectively collected specimens from a randomized clinical trial [8]. Together, these data suggest that reduced tumor cell fitness, for example, as a consequence of chemotherapy, is associated with capsule formation and that the resulting capsule represents a reparative hepatic process rather than tumor-induced “desmoplasia” in its narrower sense. We next treated mice with chemotherapy and used a genetic model to ablate established murine liver metastases. We consistently observed perimetastatic capsule formation, linking reduced tumor cell fitness to the formation of encapsulating fibrosis. Similarly, in a previous study, replacement could be converted to encapsulated growth when reducing cancer cell fitness by interfering with cell motility in a murine colorectal cancer liver metastasis model [9].

The term “encapsulated” has been used before in some studies to denote a growth pattern corresponding to the “desmoplastic” pattern as described in the current scoring guidelines [1, 10]. Based on the reasoning above, we suggest using “encapsulated” instead of “desmoplastic” in future works that address the growth patterns, and for clinical scoring algorithms.
